# Clinicoradiological Dissociation in a Giant Lytic Posterior Fossa Epidermoid Cyst: A Case Report

**DOI:** 10.7759/cureus.103911

**Published:** 2026-02-19

**Authors:** Oumaima Monadi, Hajar Hamadi, Yassine Ait M’barek, Lamia Benantar, Khalid Aniba

**Affiliations:** 1 Neurological Surgery, Ibn Tofail Hospital, Mohammed VI University Hospital, Marrakech, MAR; 2 Neurological Surgery, Mohammed VI University Hospital, Marrakech, MAR

**Keywords:** bone lysis, cholesteatoma, giant epidermoid cyst, pearly tumor, posterior cranial fossa tumor

## Abstract

Intracranial epidermoid cysts (ECs) are rare benign congenital lesions characterized by slow growth and late clinical presentation. Giant ECs of the posterior fossa are exceptionally uncommon and typically associated with early neurological deterioration due to the confined anatomy in this region. We report the case of a 74-year-old woman presenting with chronic headache and mild vertigo, with no focal motor or cranial nerve deficits. MRI revealed a giant extra-axial EC of the left posterior fossa with occipital bone lysis on CT scan. Despite the radiological findings, no acute hydrocephalus or intracranial hypertension was present. The patient underwent surgical resection. Histopathological examination confirmed the diagnosis of an EC. This case highlights the striking clinicoradiological dissociation that may be observed in giant posterior fossa ECs.

## Introduction

Intracranial epidermoid cysts (ECs) are rare, benign congenital lesions, accounting for 0.2-1.8% of all intracranial tumors and up to 7% of lesions arising in the cerebellopontine angle (CPA) [1,2]. ECs arise from ectodermal inclusions during neural tube closure between the third and fifth weeks of gestation, resulting in a cyst lined by keratinized stratified squamous epithelium that enlarges slowly through progressive epithelial desquamation [3,4]. Despite their benign histology, ECs may be responsible for significant neurological morbidity due to their predilection for eloquent intracranial spaces [5]. They most frequently involve the CPA, fourth ventricle, and the sellar/parasellar regions [1,4-6]. ECs demonstrate a slow linear growth pattern, often remaining clinically silent for years until mass effect or irritation of adjacent neural or vascular structures occurs [1].

Giant ECs are particularly uncommon, defined as lesions exceeding 5 cm in maximal diameter [4]. In the posterior fossa, such lesions are generally expected to produce early neurological manifestations given the confined anatomy and proximity to the brainstem, cranial nerves, and cerebellum. However, rare cases challenge this assumption, presenting with disproportionately mild or nonspecific symptoms despite extensive lesion size and radiological aggressiveness [1].

Here, we report a case of a giant extradural extradiploic posterior fossa EC associated with marked bone lysis, presenting with headache, mild vertigo, and no focal neurological deficit. This case highlights the striking dissociation that may exist between lesion volume, radiological severity, and clinical presentation.

## Case presentation

A 74-year-old woman presented with a four-month history of chronic headache, progressively increasing in intensity, associated with mild vertigo. There were no other accompanying symptoms such as vomiting, visual disturbances, seizures, or focal neurological deficit. Her medical history was notable for arterial hypertension.

Local physical examination revealed a painless mass on the left occipital region, without overlying skin changes or signs of inflammation. The mass was non-tender, non-pulsatile, and immobile, with a soft to firm consistency. Neurological examination was completely normal, with no signs of cranial nerve palsy, cerebellar signs, pyramidal signs, or sensory deficits. Fundoscopic eye examination was normal.

MRI of the brain showed a giant extra-axial lesion of the left posterior fossa measuring 99 x 58x 63 mm, occupying the cerebellar hemispheric region and extending inferiorly and laterally. The lesion exerted a significant mass effect on the adjacent cerebellar parenchyma, brainstem, and fourth ventricle. Despite this, there was no evidence of acute obstructive hydrocephalus, ventricular dilatation, or transependymal edema.

T1-weighted sequences showed a well-demarcated hypointense to isointense lesion with no significant enhancement following gadolinium administration. T2-weighted images showed a heterogeneously hyperintense mass, with a lobulated contour and insinuating growth pattern along cisternal spaces. On fluid-attenuated inversion recovery (FLAIR) sequences, the lesion displayed incomplete suppression, appearing heterogeneously, a characteristic feature of ECs.

**Figure 1:**
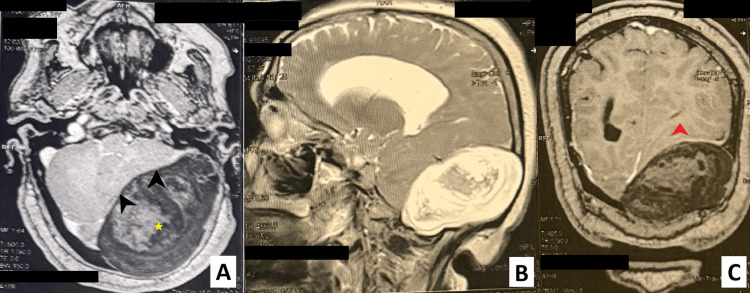
Preoperative MRI of the brain showing a massive posterior fossa lesion. A: Axial T1-weighted MRI with contrast enhancement showing a large extra-axial hypointense to isointense lesion (star) occupying the left posterior fossa, causing marked compression of the cerebellar hemisphere and brainstem (black arrow head). The lesion shows no contrast uptake after gadolinium injection. B: Sagittal T2-weighted MRI demonstrating a giant heterogeneously hyperintense mass with lobulated contours, extending inferiorly and posteriorly, with significant mass effect on the posterior fossa structures. C: Coronal T1- weighted sequence with contrast enhancement showing mass effect on the left lateral ventricle (red arrow head) with no significant hydrocephalus.

Diffusion-weighted imaging (DWI) revealed marked diffusion restriction throughout the lesion, with corresponding low signal on apparent diffusion coefficient (ADC) maps, consistent with keratinous content and strongly supporting the diagnosis of an EC (Figure 1).

**Figure 2:**
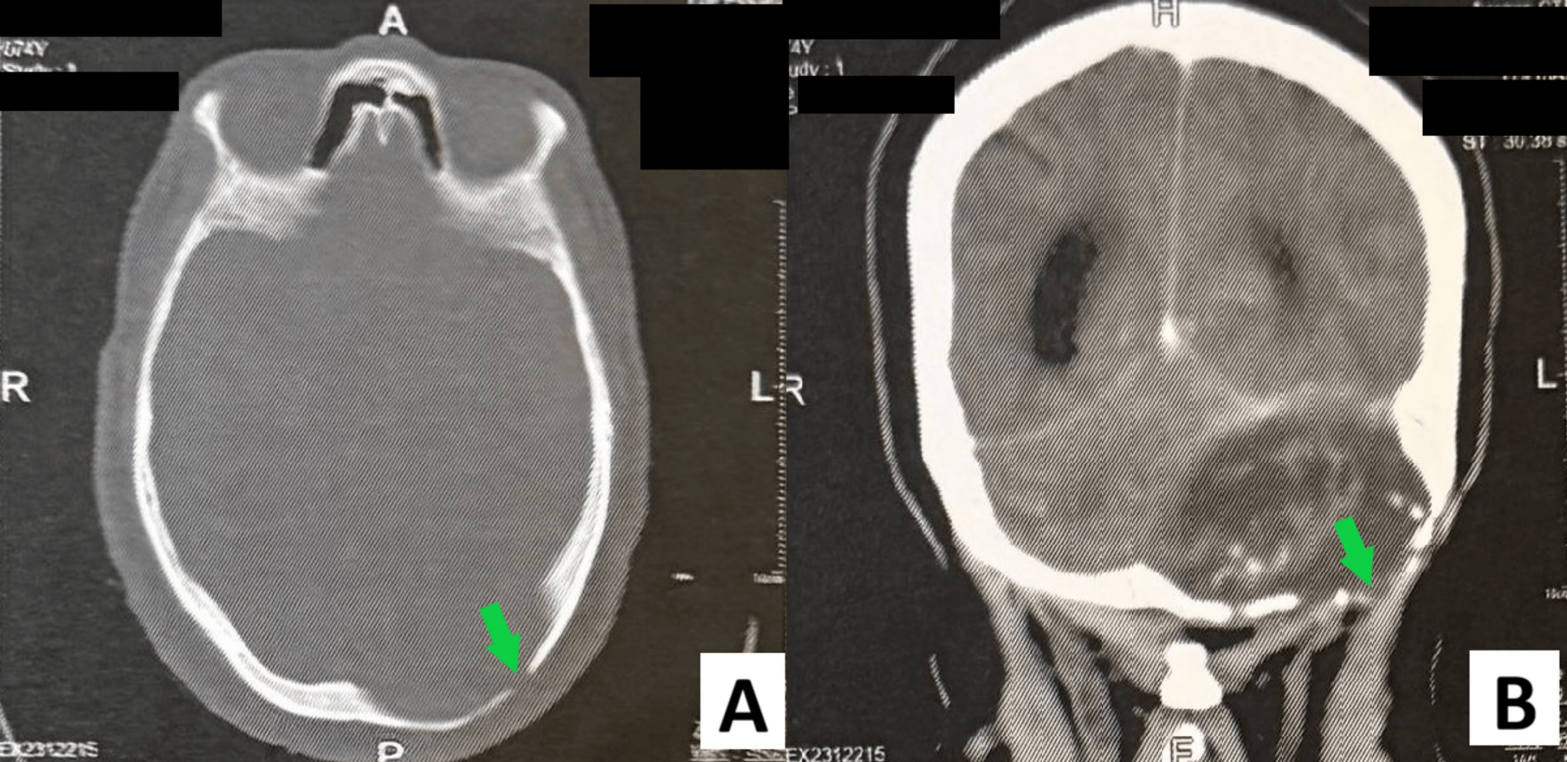
Preoperative CT scan findings. A: Axial CT scan bone reconstruction demonstrating extensive osteolysis and thinning of the left occipital bone (green arrow), consistent with a long-standing, slowly progressive lesion. B: Coronal non-contrast CT scan showing a large hypodense extra-axial lesion occupying the left posterior cranial fossa, exerting mass effect on the adjacent cerebellar hemisphere.

A complementary non-contrast CT scan of the brain was performed to further characterize the lesion. It revealed a large hypodense lesion of the left posterior cranial fossa, with well-defined but irregular margins. CT bone window demonstrated extensive osteolysis of the left occipital bone, with thinning and erosion of the diploic space. The outer table appeared expanded and remodeled rather than aggressively destroyed, consistent with a long-standing, slowly progressive process (Figure 2). Overall, radiological findings were consistent with a giant posterior fossa EC, but an intradiploic EC could not be excluded at this time.

**Figure 3:**
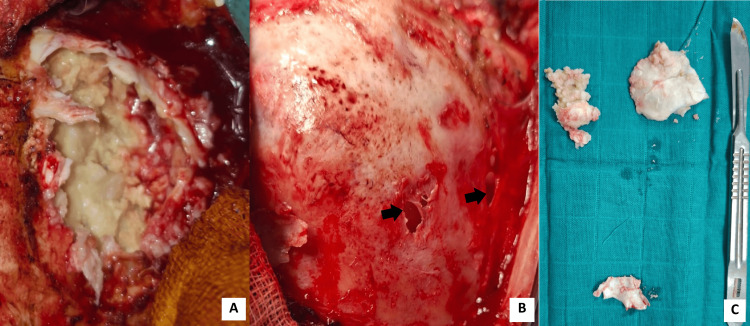
Images illustrating intraoperative findings. A: Intraoperative view after capsular opening and exposure of the lesion, showing the characteristic pearly white, friable keratinous content. B: Image highlighting the thinned and eroded occipital bone, demonstrating smooth remodeling and osteolysis (black arrows) consistent with the slow progress and growth of the cyst. C: Gross appearance of the cyst contents, consisting of lamellated, waxy keratin debris, typical of an epidermoid cyst.

The patient underwent surgical resection via a left suboccipital approach. Following skin incision and muscle dissection, marked thinning and erosion of the left occipital bone were encountered. Upon exposure, a thick capsule and abundant pearly material were visible, typical of ECs. The content was soft, waxy, and avascular, allowing progressive debulking. The lesion extended into the posterior fossa, with close proximity to the cerebellar surface and brainstem. However, the dura mater was intact with no visible invasion of the adjacent neural tissue. Careful piecemeal evacuation of the cyst contents was performed, followed by meticulous dissection of the capsule. Portions of the capsule were firmly adherent to the dura mater; therefore, subtotal capsular resection was performed in these areas to avoid the risk of neurological injury. Profuse irrigation with saline solution was performed to minimize the risk of aseptic meningitis. No intraoperative complications were encountered (Figure 3).

**Figure 4:**
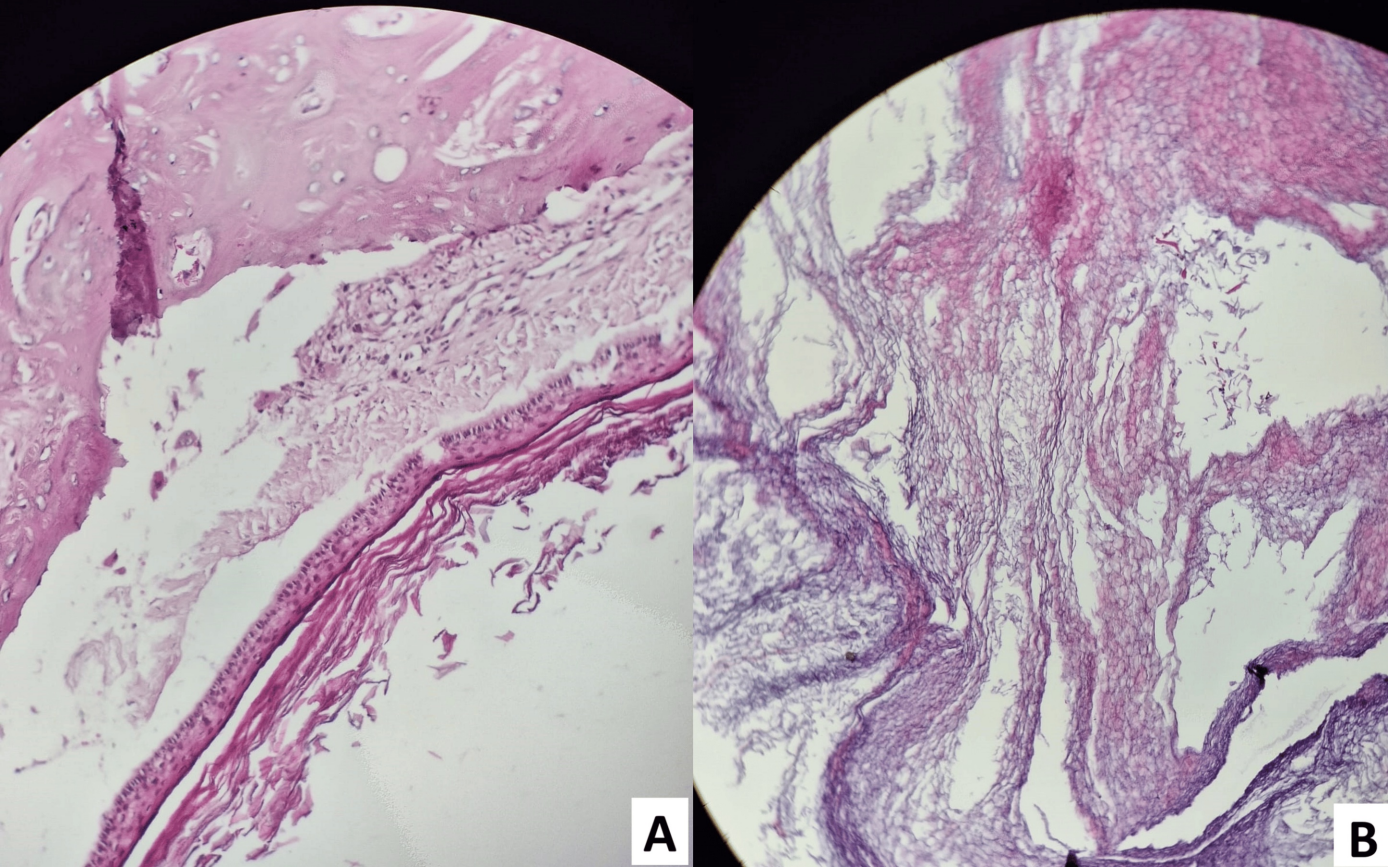
Histological sections of the resected cyst stained with hematoxylin and eosin (×40 magnification). A: Histological section showing stratified squamous epithelium, with orderly layers and no obvious cytological atypia, mitotic activity, or malignant transformation. B: Histological section demonstrating abundant lamellar orthokeratotic cystic content.

Histopathological examination revealed a cystic lesion lined by stratified keratinized squamous epithelium, with a well-preserved granular layer. The cyst lumen contained abundant lamellated keratin debris. No cytological atypia, mitotic activity, or malignant transformation was identified. These findings were consistent with an EC (Figure 4).

**Figure 5:**
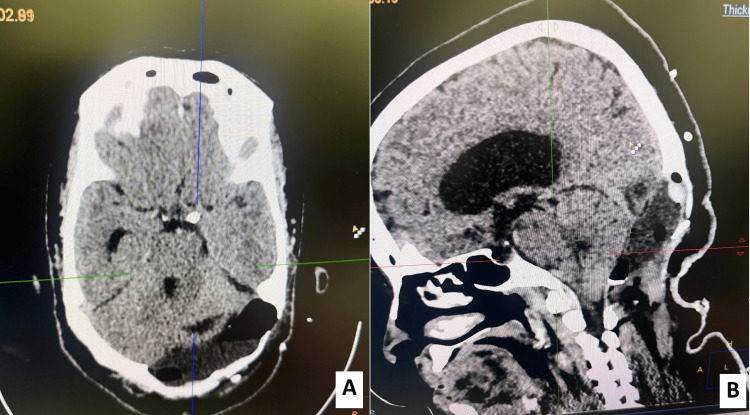
Postoperative CT scan. A: Axial non-contrast CT image showing postoperative adequate decompression of the posterior fossa and no residual mass effect, with near-complete resection of the epidermoid cyst. B: Sagittal CT reconstruction demonstrating re-expansion of the fourth ventricle and absence of acute hemorrhage or obstructive hydrocephalus, consistent with a favorable postoperative outcome.

The postoperative course was uneventful, and the patient was discharged on postoperative day four. At the three-month follow-up, she reported significant improvement in headaches and vertigo, with no clinical signs of recurrence. Control CT showed near-complete resection of the left posterior fossa EC, with satisfactory decompression of the cerebellum and brainstem. The fourth ventricle was re-expanded, and no acute hemorrhage, residual mass effect, or obstructive hydrocephalus was observed (Figure 5). Long-term follow-up with clinical assessment and control MRI was recommended at six months postoperatively, then at one year, and annually thereafter if the patient remained asymptomatic.

## Discussion

Intracranial ECs, also known as pearly tumors or cholesteatomas, are benign congenital lesions characterized by slow, linear growth and a strong tendency to remain clinically silent for prolonged periods [1,6,7]. These tumors are commonly located in the CPA, followed by occurrence in the lateral ventricles, cerebral hemispheres, basal cisterns, orbits, and, exceptionally, spine [6,7].

Giant ECs, generally defined as lesions exceeding 5 cm in diameter, represent an exceptionally rare subgroup of intracranial EC [1,4]. Their rarity is amplified when located in the posterior fossa. In a comprehensive literature review published in 2021, Spinato et al. identified only 12 reported cases of posterior fossa giant ECs [1]. The embryological timing of ectodermal cell inclusion appears to influence the anatomical compartment in which ECs develop. Early ectopic ectodermal inclusions are more likely to result in intradural lesions, whereas later inclusions tend to form extradural or intradiploic ECs [4]. Extradural ECs account for a minority of cases, of which approximately 25% are intradiploic [4].

Patients usually present with symptoms of a slowly enlarging mass, with common clinical manifestations including headache, cranial nerve deficits, cerebellar signs, and hydrocephalus [1,4,6,7]. Extracranial extension, as seen in intradiploic or extradural growth patterns, may lead to earlier detection due to a visible or palpable mass before the onset of neurological symptoms [4]. This explains the prolonged and heterogeneous symptom duration ranging from months to several years [1,4]. This is consistent with our case, with a remarkable discrepancy between the exceptional size of the lesion and the paucity of neurological symptoms. Despite measuring nearly 10 cm in its greatest dimension, exerting significant mass effect on the cerebellum, brainstem, and fourth ventricle, and being associated with extensive occipital bone lysis, the patient presented only with chronic headaches and mild vertigo, without focal neurological deficits or signs of raised intracranial pressure.

MRI is the imaging modality of choice, with CT complementing assessment of bone involvement [4,5]. Typical features include a lesion that is hypointense on T1, hyperintense on T2, shows incomplete suppression on FLAIR, and demonstrates marked diffusion restriction on DWI, which is considered pathognomonic [1,4,5]. A CT scan is useful for assessing bone involvement, particularly in cases with intradiploic or extradural extension [4,5].

Surgery is universally regarded as the treatment of choice for intracranial ECs [3,5,7,8]. As the squamous stratified epithelium that constitutes the capsule is the real disease, complete removal of the cyst contents and capsule is acknowledged to be the primary goal of the treatment [4,6,8]. However, when the capsule is adherent to adjacent neurovascular structures, and given the high risk of irreversible neurological deficits, a more conservative approach is preferred [6-8]. In our patient, the capsule was firmly adherent to the dura mater, which prompted a subtotal resection of the capsule to avoid postoperative neurological deficits.

Morbidity and mortality in patients with ECs have dramatically decreased [7,8]. The most frequent surgical complications are aseptic meningitis and cranial nerve deficits, which can be avoided or decreased by avoiding dissection of the capsule when it adheres to neurovascular structures [7,8].

Long-term follow-up is essential, particularly after subtotal resection, as recurrence is primarily related to residual capsular tissue [7,8]. Recurrence may occur years or even decades after surgery, with reported rates varying widely in the literature [8]. Imaging should be performed at three and six months postoperatively, followed by annual assessments [7,8]. Extended follow-up beyond 10 years may be warranted due to the slow-growing nature of ECs and their potential for delayed recurrence [7]. Complete resection is associated with the lowest recurrence risk, whereas intentionally preserved capsule adherent to critical neurovascular structures remains the main source of regrowth [7,8].

## Conclusions

GECs of the posterior fossa are exceptionally rare lesions and may present with a striking clinicoradiological dissociation, as illustrated in our case. This underscores the indolent nature and adaptive growth of ECs, which can delay diagnosis even in anatomically constrained regions. Surgical management should prioritize maximal safe resection, balancing effective decompression with preservation of neurological function. Long-term radiological follow-up remains essential given the potential for late recurrence.
